# Vertebral body defects treated with umbilical-cord mesenchymal stem cells combined with hydroxyapatite scaffolds: The first case report

**DOI:** 10.1016/j.ijscr.2019.12.002

**Published:** 2019-12-12

**Authors:** Ahmad Jabir Rahyussalim, Muhammad Deryl Ivansyah, Ahmad Nugroho, Rio Wikanjaya, Anissa Feby Canintika, Tri Kurniawati

**Affiliations:** a Department of Orthopaedics and Traumatology, Faculty of Medicine, Universitas Indonesia-Cipto Mangunkusumo Hospital, Jakarta, Indonesia; b Divion of Spine, Department of Orthopaedics and Traumatology, Faculty of Medicine, Universitas Indonesia-Cipto Mangunkusumo Hospital, Jakarta, Indonesia; c Stem Cell Integrated Service Unit, Cipto Mangunkusumo Hospital, Jakarta, Indonesia

**Keywords:** Vertebral body defects, Mesenchymal stem cells, Hydroxyapatite

## Abstract

**Introduction:**

Vertebral body defects (VBDs) are one of the most frequent orthopaedic disorders. Such defects often require bone grafts or fusion procedures; however, both procedures often fail due to various factors. Mesenchymal stem cells (MSCs) have been used as a potential therapy to fill bone voids in bone defects, and they may be a potential treatment for VBDs. We reported VBDs treated with MSCs combined with hydroxyapatite scaffolds.

**Presentation of case:**

A 27-year-old female presented with recurrent back pain. She had a history of decompression and stabilization procedure one year ago after diagnosed with spinal tuberculosis. Initially, she felt back pain that intensifies with activity and relieved with rest. She noticed that the pain begun when once she heard a crack sound on her back while trying to get up from sitting position. There was no history of numbness or tingling sensation. There were no walking problems. Other functions, including micturition and defecation, were within normal limits. The patient firstly underwent lumbotomy procedure, and the images were all confirmed with fluoroscopy X-ray. The vertebrae went debridement, and finally, the bone defect was filled with 20 millions of umbilical cord-mesenchymal stem cells (UC-MSCs) combined with hydroxyapatite in 2 cc of saline.

**Discussion:**

At three months postoperative, the patient could walk and had no pain. At six months of follow-up, no complications occurred. We also did not see any signs of neoplasm formation, which is consistent with previous studies that used MSCs for orthopaedic treatment. Moreover, no significant bone deformation or spinal cord compression was observed, which suggested the safety of the transplantation procedure.

**Conclusions:**

We found that MSCs combined with hydroxyapatite represents a potential therapy for bone regeneration in VBD. Further clinical studies are required to investigate the safety and efficacy of this combination of therapy in VBDs.

## Highlights

• Case report of patient with vertebral body defects which treated by combination of mesenhymal stem cell and hydroxyapatite.
• Mesenchymal stem cell combined wiht hydroyapatite have potential therapy for vertembral body defect.
• Further larger studies with longer duration of follow up are requierd to investigate the safety and efficacy.

## 1 Introduction

Vertebral body defects (VBDs) represent one of the most common orthopaedic disorders, and they can lead to spinal instability and disturbance of surrounding tissues [1]. Often, spinal tuberculosis results in these defects. These defects often require bone grafts or fusion procedures; however, approximately 25–40% of vertebral fusion procedures fail to heal due to surgical technique (strength of fixation, soft tissue trauma) or host factors that impede healing like tobacco abuse [[2], [3], [4], [5]]. Osteoconductive scaffolds have a history as graft extenders to help bridge gaps between bones while reducing the need for autologous bone tissue. The most abundant of these materials are prepared from cadaveric bone tissue, but frozen materials present contamination issues, and extensively processed allograft has reduced efficacy [6]. Various synthetic scaffolds have been developed, and although these products mimic some of the characteristics of bone, their effectiveness has proven highly variable [[7], [8], [9]]. This indicates the need for efficacious therapies that can fill bone voids in VBDs appropriately.

As a standard surgical treatment, autologous bone grafts are used to promote healing of the vertebral body. However, graft harvesting is associated with numerous drawbacks; these include donor-site morbidity, limited bone quantity, and variable bone quality [[10], [11], [12]]. On the other hand, allografts are associated with immune rejection and the risk of infectious diseases. Therefore, it is a challenge to find an alternative treatment strategy for VBDs. Often, synthetic calcium-based bone materials are used for treating bone defects due to their similarity to the mineral phase of bone, their osteoconductivity and good biocompability [13,14]. Mesenchymal stem cells (MSCs) have been studied as a potential therapeutic tool for bone tissue regeneration. The osteogenic and immunological properties of MSCs, together with the possibility to relatively easily obtain, cultivate and produce these cells in large amounts, represent advantages over other types of cells. MSCs are particularly attractive for their low immunogenicity [[15], [16], [17]]. Combined with hydroxyapatite scaffolds, they enhanced the osteoinductivity of calcium-based scaffolds and promoted bone healing in various experimental bone defects including long bone fractures [18], spinal fusion [19] and craniofacial defects [[20], [21], [22]]. Nevertheless, reports discussing the effect of MSCs in the treatment of VBDs are scarce in the literature, although this approach represents a promising tool for vertebral bone regeneration [10]. To date, there are only animal studies regarding the use of MSCs in treating vertebral body defects. We reported a 27-year-old female with VBD treated with MSCs combined with hydroxyapatite scaffolds. The work has been reported in line with the SCARE criteria [23].

## 2 Case report

**Fig. 1:**
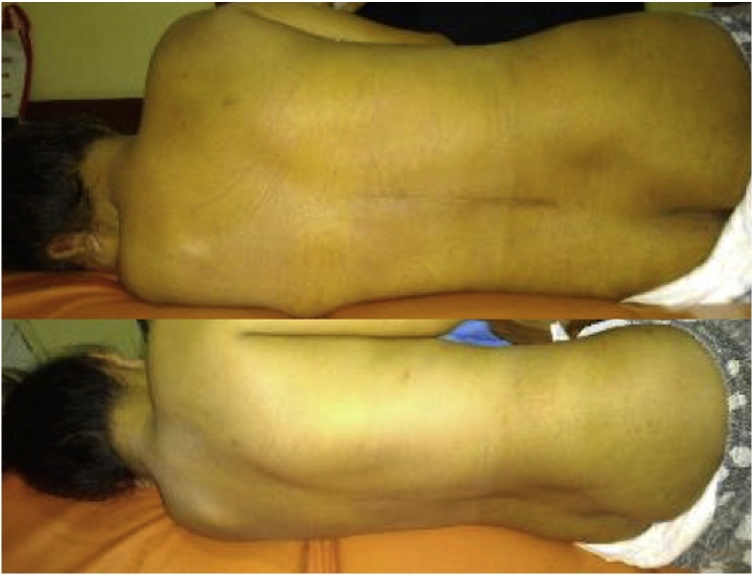
Clinical picture shows scar on thoracolumbar area.

**Fig. 2:**
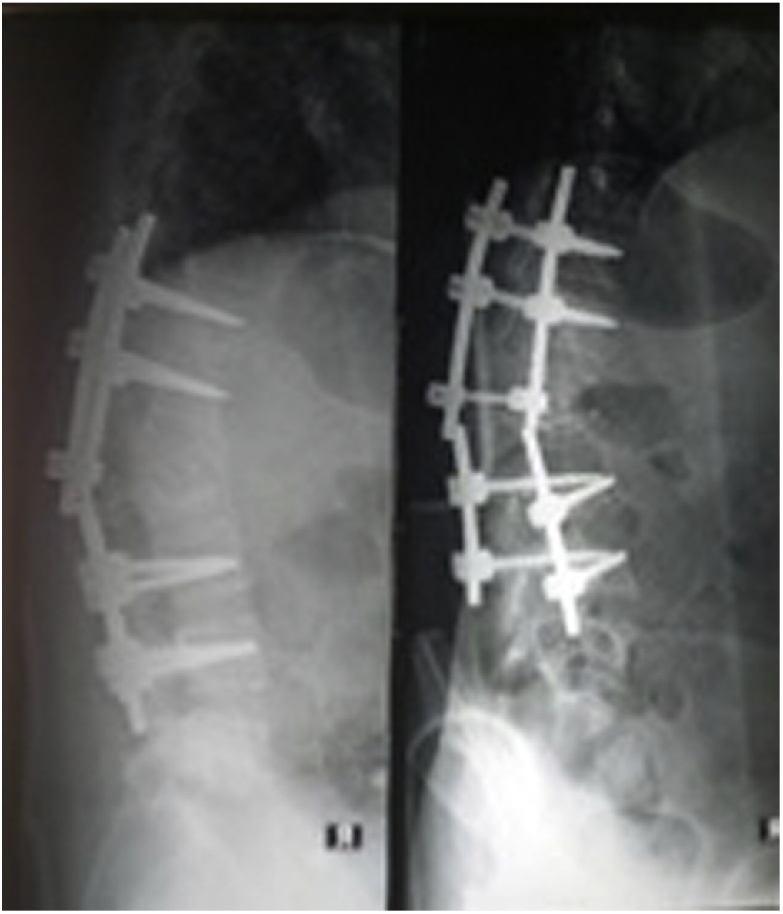
Broken of bilateral rod.

**Fig. 3:**
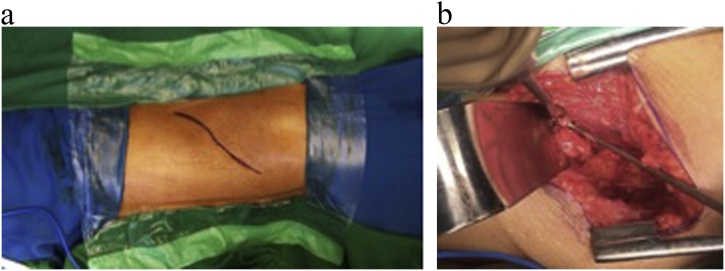
Lumbotomy procedure on left side. It shows the vertebral body defect at thoracolumbar area.

**Fig. 4:**
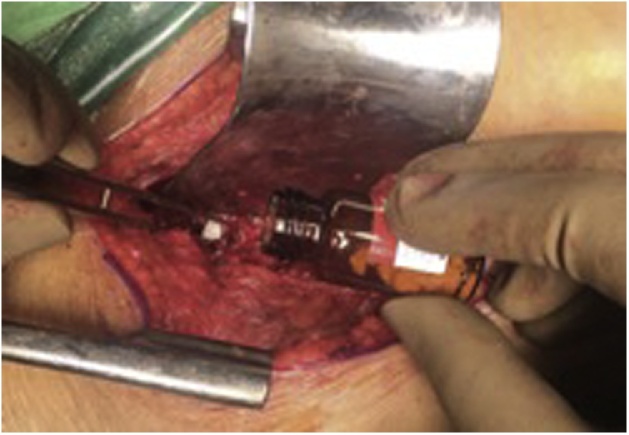
Procedure of putting hydroxy apatite with stem cell on vertebral body defect.

**Fig. 5:**
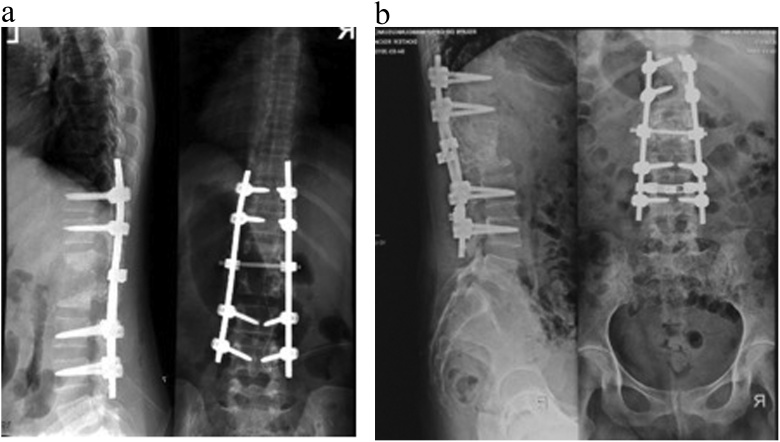
(a) This picture shows intact implant on posterior and defect big vertebral defect at level L1 L2; (b) Bone subtitutes with mesenchymal stem cell are on the defect of vertebral body. They fulfilled the defect on good position.

A 27-year-old female presented with recurrent back pain. She had history of decompression and stabilization procedure one year ago after diagnosed with spinal tuberculosis. Initially, she felt back pain that intensifies with activity and relieved with rest. She noticed that the pain begun when once she heard a crack sound on her back while trying to get up from sitting position. There was no history of numbness or tingling sensation. There were no walking problems. Other functions, including micturition and defecation, were within normal limits (Fig. 1, Fig. 2, Fig. 3, Fig. 4, Fig. 5).

Approximately one year ago the patient had surgery due to tuberculosis of the spine of L1–L2 and received rifampicin, isoniazide, pyrazinamide, and ethambutol. After one and a half month, she discontinued the drugs by herself. Physical examination demonstrated surgical scar. Any other past history of medical and drug history were denied. On palpation, there were mild tenderness on L1–L2 with visual analog scale (VAS) of 3–4. She could not move her back freely due to pain. Neurological examination demonstrated unremarkable findings. Series of X-ray examinations demonstrated slight kyphotic alignment of the bone, yet a broken metal rod on both sides of the bar. The L1 and L2 vertebrae shows solid fusion mass on L1 and L2. No endplate sclerosis nor disc space narrowing. Laboratory findings, including complete blood count, C-reactive protein, kidney and liver function tests, and blood coagulation tests, were within normal limits.

The patient firstly underwent lumbotomy procedure, and the images were all confirmed with fluoroscopy X-ray. The vertebrae went debridement, and finally, the bone defect were filled with 20 millions of umbilical cord-mesenchymal stem cells (UC-MSCs) combined with hydroxyapatite in 2 cc of saline. The sample of UC-MSCs was pre-characterised human UC-MSCs at passage 4, which was isolated by the multiple harvest explant methods, cryopreserved, and stored in liquid nitrogen. The sample was obtained from Stem Cell Medical Technology Service Unit, Cipto Mangunkusumo Hospital, Jakarta, Indonesia. Cryopreserved UC-MSCs were thawed rapidly and recultured in a 25 cm^2^ tissue culture (T25) flasks in 10 % platelet lysate containing complete medium as previously described. At 80–90% confluence, the cells were harvested and counted [24].

## 3 Discussion

Vertebral body defects represent one of the most common orthopaedic disorders. Often, spinal tuberculosis results in these defects. Depending on the type and severity of the injury, both conservative and/or surgical treatment is applied. Severe VBDs requiring vertebral body replacement by an autologous graft or vertebroplasty occur mainly due to (1) traumatic comminute fractures, (2) compressive fractures due to osteoporosis, (3) vertebral osteolytic malignant tumour metastases or (4) vertebral hemangioma. Traumatic comminute fractures affect mostly young people and often have a severe impact on the integrity of the spinal column, in a significant number of cases complicated by spinal cord injury. Moreover, the integrity of the spine can be disrupted by other pathological processes, for example metastatic foci of malignant tumours or metabolic disorders (osteoporosis, hyperparathyroidism, Paget disease) [10]. In this case, the defect was caused by tuberculosis of the spine.

In this report, we treated the patient with MSCs in combination with a hydroxyapatite-based scaffold (Bongros®). We have found that at three months postoperative, the patient could walk and had no pain. At six months of follow-up, no complications occurred. We also did not see any signs of neoplasm formation, which is consistent with previous studies that used MSCs for orthopaedic treatment [10,25]. Also, no significant bone deformation or spinal cord compression was observed, which suggested the safety of the transplantation procedure. To date, there are no studies regarding the use of MSCs combined with hydroxyapatite in human. Vanecek et al. [10] evaluated the effect of MSCs in combination with a hydroxyapatite-based scaffold (CEM-OSTETIC®) on bone regeneration in rat models with VBDs. There were four groups: (1) rats with a VBD only (group 1) (n = 8); (2) rats with a VBD and with an implanted hydroxyapatite scaffold (group 2) (n = 7); (3) rats with a VBD with a hydroxyapatite scaffold as well as 0.5 × 10^6^ human MSCs implanted (group 3) (n = 7); (4) rats with a VBD and with a hydroxyapatite scaffold as well as 5 × 10^6^ human MSCs implanted (group 4) (n = 8). They have observed the significant formation of bone tissue in the defect in animals receiving a hydroxyapatite bone scaffold loaded with 5 million MSCs. The scaffold was incorporated in the bone tissue and partially resorbed.

Previously, we conducted an experimental study using a rabbit spondylitis model. We treated six rabbits using bone marrow-derived mesenchymal stem cells (BM-MSCs) and antituberculous drugs [1]. The rabbits were divided into two groups: rabbits which was positive for tuberculosis of the spine by culture, polymerase chain reaction (PCR), and histopathologically (intervention group) (n = 3), and (2) rabbits which was positive for tuberculosis of the spine by polymerase chain reaction (PCR) and histopathologically (controls). The intervention group received filled with 150 mg hydroxyapatite and transplanted with 6 × 10^6^ BM-MSCs. After six weeks we found that the ossification score in the intervention group exceeded 30, while the score of the controls was 25.67 [1]. Due to their properties, MSCs represent a potential tool to improve the healing of bone defects in orthopaedics [10]. The mechanisms by which the transplanted cells contribute to bone formation are still not completely understood. Huang et al. [19] showed that MSCs combined with a hydroxyapatite/PLGA/collagen I scaffold differentiate into osteoblasts and produce extracellular matrix within the graft for posterolateral spinal fusion. Other authors have shown that transplanted MSCs associated time after transplantation and recruit cells from the host tissue, thus contributing increased bone formation and bone healing somewhat indirectly [26].

Due to their osteogenic potential and immunomodulatory, anti-inflammatory and anti-apoptotic properties, MSCs have the potential to be used as the primary treatment for diseases affecting bone tissues [27]. Various animal models have also demonstrated that MSCs accelerate and promote new bone formation. Factors secreted by MSCs include trophic and immunomodulatory factors [28]: insulin-like growth factor-1 (IGF-1) induces osteoblast proliferation and differentiation [29], and vascular endothelial growth factor (VEGF) promotes angiogenesis. VEGF also contributes to osteogenesis by enhancing the survival and differentiation of endothelial cells [30]. TGF-β1 stimulates migration of osteoprogenitor cells, and it regulates cell proliferation and differentiation. Moreover, it enhances extracellular matrix (ECM) production [31]. All of these factors, secreted by MSCs, contribute to the bone formation process.

## 4 Conclusions

We found that MSCs combined with hydroxyapatite represents a potential therapy for bone regeneration in VBD. Further clinical studies are required to investigate the safety and efficacy of this combination of therapy in VBDs.

## Funding

Higher Education of Applied Research (No. 463/UN2.R3.1/HKP05.00/2018).

## Ethical approval

Ethical approval was received from the Ethical Committee of Faculty of Medicine, Universitas Indonesia (No. 0489/UN2.F1/ETIK/2018).

## Consent

Written informed consent was obtained from the patient for publication of this case report and accompanying images. A copy of the written consent is available for review by the Editor-in-Chief of this journal on request.

## Author contribution

Ahmad Jabir Rahyussalim: performing the procedure, study concept, data collection.

Muhammad Deryl Ivansyah: performing the procedure, data collection, writing the paper.

Ahmad Nugroho: data collection, writing the paper.

Rio Wikanjaya: data collection.

Anissa Feby Canintika: writing the paper.

Tri Kurniawati: processing the stem cells, writing the paper.

## Registration of research studies

This study has been registered at researchregistry.com (UIN: researchregistry5169).

## Guarantor

Ahmad Jabir Rahyussalim.

## Provenance and peer review

Not commissioned, externally peer-reviewed.

## Declaration of Competing Interest

None.

## References

[1] Rahyussalim A.J., Kurniawati T., Siregar N.C. (2016). New bone formation in tuberculous-infected vertebral body defect after administration of bone marrow stromal cells in rabbit model. Asian Spine J..

[2] Tzioupis C., Giannoudis P.V. (2007). Prevalence of long-bone non-unions. Injury.

[3] Boden S.D. (2000). Biology of lumbar spine fusion and use of bone graft substitutes: present, future, and next generation. Tissue Eng..

[4] Green E., Lubahn J.D., Evans J. (2005). Risk factors, treatment, and outcomes associated with nonunion of the midshaft humerus fracture. J. Surg. Orthop. Adv..

[5] Kanakaris N.K., Giannoudis P.V. (2007). The health economics of the treatment of long-bone non-unions. Injury.

[6] Ibrahim T., Stafford H., Esler C.N.A., Power R.A. (2004). Cadaveric allograft microbiology. Int. Orthop..

[7] Kao S.T., Scott D.D. (2007). A review of bone substitutes. Surg. Clin. North Am..

[8] Shegarfi H., Reikeras O. (2009). Review article: bone transplantation and immune response. J. Orthop. Surg. (Hong Kong).

[9] Clough B.H., McCarley M.R., Krause U. (2015). Bone regeneration with osteogenically enhanced mesenchymal stem cells and their extracellular matrix proteins. J. Bone Miner. Res..

[10] Vaněček V., Klíma K., Kohout A. (2013). The combination of mesenchymal stem cells and a bone scaffold in the treatment of vertebral body defects. Eur. Spine J..

[11] Rogers G.F., Greene A.K. (2012). Autogenous bone graft: basic science and clinical implications. J. Craniofac. Surg..

[12] Kloss F.R., Offermanns V., Kloss-Brandstätter A. (2018). Comparison of allogeneic and autogenous bone grafts for augmentation of alveolar ridge defectsäA 12-month retrospective radiographic evaluation. Clin. Oral Implants Res..

[13] Wang Z., Lu B., Chen L., Chang J. (2011). Evaluation of an osteostimulative putty in the sheep spine. J. Mater. Sci. Mater. Med..

[14] Bauer T.W., Muschler G.F. (2000). Bone graft materials: an overview of the basic science. Clin. Orthop. Relat. Res..

[15] Kim H.J., Park J.B., Lee J.K. (2008). Transplanted xenogenic bone marrow stem cells survive and generate new bone formation in the posterolateral lumbar spine of non-immunosuppressed rabbits. Eur. Spine J..

[16] Kim J., Hematti P. (2009). Mesenchymal stem cell-educated macrophages: a novel type of alternatively activated macrophages. Exp. Hematol..

[17] Ghannam S., Bouffi C., Djouad F., Jorgensen C., Noël D. (2010). Immunosuppression by mesenchymal stem cells: Mechanisms and clinical applications. Stem Cell Res. Ther..

[18] Choi H.J., Kim J.M., Kwon E. (2011). Establishment of efficacy and safety assessment of human adipose tissue-derived mesenchymal stem cells (hATMSCs) in a nude rat femoral segmental defect model. J. Korean Med. Sci..

[19] Huang J.W., Lin S.S., Chen L.H. (2011). The use of fluorescence-labeled mesenchymal stem cells in poly(lactide-co-glycolide)/hydroxyapatite/collagen hybrid graft as a bone substitute for posterolateral spinal fusion. J. Trauma – Inj. Infect. Crit. Care.

[20] Miura M., Miura Y., Sonoyama W., Yamaza T., Gronthos S., Shi S. (2006). Bone marrow-derived mesenchymal stem cells for regenerative medicine in craniofacial region. Oral. Dis..

[21] Liang H., Wang K., Shimer A.L., Li X., Balian G., Shen F.H. (2010). Use of a bioactive scaffold for the repair of bone defects in a novel reproducible vertebral body defect model. Bone.

[22] Zhu X.S., Zhang Z.M., Mao H.Q. (2011). A novel sheep vertebral bone defect model for injectable bioactive vertebral augmentation materials. J. Mater. Sci. Mater. Med..

[23] Pawitan J.A., Kispa T.D., Liem I.K., Mujadid F., Novialdi N. (2018). The potential of basal medium astemporary prolong culture of umbilical cord derived mesenchymalstem cells. Biomed. Pharmacol. J..

[24] Centeno C., Schultz J., Cheever M., Robinson B., Freeman M., Marasco W. (2010). Safety and complications reporting on the re-implantation of culture-expanded mesenchymal stem cells using autologous platelet lysate technique. Curr. Stem Cell Res. Ther..

[25] Boukhechba F., Balaguer T., Bouvet-Gerbettaz S. (2011). Fate of bone marrow stromal cells in a syngenic model of bone formation. Tissue Eng. – Part A..

[26] Linero I., Chaparro O. (2014). Paracrine effect of mesenchymal stem cells derived from human adipose tissue in bone regeneration. PLoS One.

[27] Ranganath S.H., Levy O., Inamdar M.S., Karp J.M. (2012). Harnessing the mesenchymal stem cell secretome for the treatment of cardiovascular disease. Cell Stem Cell.

[28] Li Y., Yu X.Y., Lin S.G., Li X.H., Zhang S., Song Y.H. (2007). Insulin-like growth factor 1 enhances the migratory capacity of mesenchymal stem cells. Biochem. Biophys. Res. Commun..

[29] Kaigler D., Krebsbach P.H., Polverini P.J., Mooney D.J. (2003). Role of vascular endothelial growth factor in bone marrow stromal cell modulation of endothelial cells. Tissue Eng..

[30] Bostrom M.P.G., Asnis P. (1998). Transforming growth factor beta in fracture repair. Clin. Orthop. Relat. Res..

[31] Bostrom M.P.G., Asnis P. (1998). Transforming growth factor beta in fracture repair. Clin. Orthop. Relat. Res..

